# Animal Agriculture and Climate Change in the US and UK Elite Media: Volume, Responsibilities, Causes and Solutions

**DOI:** 10.1080/17524032.2020.1805344

**Published:** 2020-09-07

**Authors:** Silje Kristiansen, James Painter, Meghan Shea

**Affiliations:** a College of Environmental Science and Forestry, State University of New York, Syracuse, NY, USA; b Reuters Institute for the Study of Journalism, University of Oxford, Oxford, UK; c Emmett Interdisciplinary Program in Environment and Resources, Stanford University, Stanford, CA, USA

**Keywords:** Animal agriculture, climate change, US and UK media, meat consumption, content analysis

## Abstract

Animal agriculture is a major producer of greenhouse gas emissions, equivalent to 14.5% of global emissions, which is approximately the same size as the transportation sector. Global meat consumption is projected to grow, which will increase animal agriculture’s negative impact on the environment. Public awareness of the link between animal food consumption and climate change is low; this may be one of many obstacles to more effective interventions to reduce meat consumption in Western diets, which has been proposed by many research institutions. This study analyzes how much attention the UK and US elite media paid to animal agriculture’s role in climate change, and the roles and responsibilities of various parties in addressing the problem, from 2006 to 2018. The results of the quantitative media content analysis show that during that period, volume of coverage remained low, and that when the issue was covered, consumer responsibility was mentioned more than that of governments or large-scale livestock farms. In similar fashion, a range of options around personal dietary change was far more prominent in the media discussion of solutions than government policies, reforming agricultural practices or holding major animal food companies accountable for their emissions.

## Introduction

The earth’s changing climate is likely to alter living conditions on this planet dramatically and sooner than expected. The 1.5-degree Intergovernmental Panel on Climate Change (IPCC) report emphasized the urgency to act (IPCC, 2018), and suggested policy options that could be taken to reduce the likelihood of severe adverse impacts to livelihoods and the environment. One of these policies is to limit the intake of animal products, or even omit them from our diet, as their production is a major cause of greenhouse gas emissions (GHGs). The estimates of the global impact of animal agriculture are that it represents 14.5% of human-induced emissions, which is approximately the same size as the emissions from the global transport sector (Bailey et al., 2014; Food and Agriculture Organization of the United Nations [FAO], 2006, 2018), a comparison often picked up by other reports (Bailey et al., 2014). This number will increase as the human population and the appetite for meat grows, particularly in China and other Asian countries (Godfray et al., 2018). The FAO expects total global meat consumption to increase by more than 70% by 2050 (FAO, 2011).

Animal products have been estimated to contribute more to GHGs, deforestation, ocean acidification, biodiversity loss, and unhealthy humans, than plant-based foods (Poore & Nemecek, 2018). Hence, part of the solution to emit less GHG is to limit the demand for GHG intensive foods through encouraging shifts to more sustainable diets (Harwatt et al., 2017; Poore & Nemecek, 2018; Wynes & Nicholas, 2017). There is a significant potential in a shift to plant-based foods – meat-eating is calculated to contribute almost four times as much to GHG emissions as a plant-based diet (Poore & Nemecek, 2018). Beside individual dietary changes other actions need to be taken too, such as reducing food loss and waste, and improving agricultural technologies and management (Springmann et al., 2018).

Wellesley et al. (2015) point to the importance of government intervention to address overconsumption of unsustainable foods. They argue the potential public backlash towards governmental involvement has been overestimated. Godfray et al. (2018) discuss a range of policy options, including the tension between state-sponsored intervention in the food system and free trade, fiscal measures, and different interventions (such as labeling) which aim to affect people’s food purchasing and consumption practices. Wynes and Nicholas (2017) found that government recommendations to the public in Australia, Canada, EU and the US tended to focus more on lower-impact actions to reduce your carbon footprint, e.g. use reusable bags instead of single use plastic bags, as these actions might be easier to engage in and less politically contested. Wynes and Nicholas also state that higher impact actions, such as a plant-based diet, may be politically less popular, and therefore avoided. The authors mention that using reusable bags instead of single use plastic bags is less than 1% effective compared to going a year without eating meat. Wynes and Nicholas say that a plant-based diet saves 0.8 tCO_2_e (tons of carbon dioxide equivalent) per year (2017, p. 4); to contrast this, they quote calculations found in Dickinson et al. (2009) showing that using reusable bags only saves approximately 5kgCO_2_e.

Despite the abundance of academic studies and NGO reports on animal agriculture’s impact on the climate, and policy options for helping reducing emissions, these seem to have had little impact, if any, on policy implementation at the international and national level. One study speaks about a “livestock policy vacuum” in which little attention is directed to the issue by policy-makers and opinion leaders (Bailey et al., 2014).

Whereas many people are aware of the GHG emissions of the energy and transportation sectors, there is an awareness gap when it comes to animal agriculture’s GHG emissions (Happer & Wellesley, 2019). Bailey et al. (2014) found that in a survey of 1000 participants each in twelve countries (including the US and UK), public recognition of the livestock sector as a contributor to climate change was markedly the lowest compared to other contributing sectors. According to their study, “over twice as many respondents identified direct transport emissions as a major contributor as identified meat and dairy production (64% vs 29%), even though the contribution to overall emissions is almost equal between the two sectors” (Bailey et al., 2014, p. 19).

In general, the gap between awareness of environmental issues and behavior change has been well-monitored (Kollmuss & Agyeman, 2002). In the specific area of dietary change, considerable research shows that simply giving people information about the health, environmental and animal welfare consequences of eating meat does not by itself reduce people’s intentions to eat less meat (Bianchi et al., 2018). Cultural norms and other structural barriers stand in the way of even knowledgeable individuals changing their diet or adopting other carbon-reduction actions (Wynes & Nicholas, 2017).

However, other research suggests that as a minimum, knowledge of an issue is a requirement, and the public cannot be expected to act on a problem without it (Neff et al., 2009). In survey work with consumers in the Netherlands and the US, the willingness of individuals to eat less meat increased with the perceived effectiveness of this action – which suggested a need for policy makers to actively inform the public more of this effectiveness (de Boer et al., 2016); and although the causal relationships between media coverage and public awareness, opinion, and willingness to change behavior are complex, in certain contexts traditional media can play a central role in setting the agenda and shaping public interest in which issues are important to think about, including the link between climate change and consumption choices (Happer & Wellesley, 2019).

With this in mind, this study provides an up-to-date and detailed context for a greater understanding of media treatments of animal agriculture and climate change, and of the ways media messages help to shape the range of arguments and perspectives informing the public debate, and how audiences may react and act on this information. We therefore focus on how much attention the US and UK elite media pay to animal agriculture’s impact on the climate, and when they do, how they portray the issue: which causes, solutions and responsible actors do they discuss?

### Media coverage and audiences

Compared to studies on climate change media coverage in general, little is known about how the media cover animal agriculture’s impact on the climate, especially in recent years. Traditional media are important and trusted agents by the public in the production, reproduction, and transformation of the meaning of news in general and environmental issues in particular (Carvalho, 2007). For example, the amount of media attention a specific issue attracts has an important “agenda-setting” effect on audience members; the more attention an issue gets, the more likely it is to seem important to an audience. It can also affect the awareness and knowledge of the general public about an issue (Schmidt et al., 2013), and awareness of an issue can be a prerequisite for the public to take action (Neff et al., 2009). With regard to climate change specifically, many people receive their information mainly from media reporting (Schäfer, 2015; Stamm et al., 2000) and media reporting, in turn, often shapes public perceptions of climate change (Liu et al., 2011; Sampei & Aoyagi-Usui, 2009).

For example, a study on meat consumption and the media based on work with focus groups in the UK, US, China and Brazil found that people’s perceptions and beliefs on climate change are culturally specific, but “are contextualized within individually constructed media environments” (Happer & Wellesley, 2019). Amongst the researchers’ findings was that although there was very low existing level of public awareness about meat consumption and climate change, there was significant potential to develop a positive narrative around the benefits of dietary change; secondly, despite the rise in social media attention, traditional media outlets exerted an important influence on potential individual and government-driven solutions. The little media attention directed to the connection between animal agriculture and the climate can be interpreted by the public as it not being that important. Therefore, traditional media remain significant in setting people’s agenda of which issues are important to think about (Happer & Wellesley, 2019).

The media historically have given the issue very little attention. One study looking at Los Angeles Times’ coverage from 1999 to 2010 found that only 5% of the 380 articles that discussed livestock addressed its connection to climate change. The most discussed issue was health, which occurred in 43% of the articles (Lee et al., 2014). Another study took a different approach and looked at meat-related articles in the New York Times from 1983 to 2011; they found that the environment was a topic mentioned in only 9% of the over 12,000 articles that were written about meat. Cuisine (35%) and economy (32%) were the most discussed topics (Chiles, 2017). Neff et al. (2009) looked at sixteen leading US newspapers from September 2005 to January 2008 and identified over 4500 articles that covered climate change. Only in 2.4% of those was the contribution of food or animal agriculture to climate change mentioned. The authors did find an increase in coverage of the issue over time, but as they argue, not enough to reflect the growing evidence of the importance of animal agriculture’s effects on the climate.

Outside of the US, three major Brazilian newspapers showed very little coverage in the years 2007–2008; in only 0.14% of the analyzed articles was animal agriculture mentioned in the context of climate change (Lahsen, 2017). According to the author, the figures for Brazil were significantly lower than other countries, even though beef is the single largest source of national emissions, a reality not shared with other countries. Almiron and Zoppeddu (2015) were able to show that the top Spanish and Italian newspapers hardly covered animal agriculture’s impact on the climate in the years 2006–2013. A qualitative study of a very limited number of articles (*n* = 9) published in the UK and US after the FAO released its Livestock’s Long Shadow report in 2006, shows that the media (unsurprisingly given the news peg) did make the link between livestock production and climate change (Kiesel, 2010). However, some commentators have pointed out that the articles showed a very cautious and voluntary approach to addressing the problem (Almiron & Zoppeddu, 2015).

We know even less about how information about animal agriculture’s impact on climate change is consumed, exchanged, and commented on via social media, despite the evidence of their huge importance as a source of information in many countries (Newman et al., 2017). We do know that some Facebook users in Sweden try to legitimize livestock production. One study looked at Facebook comments on two most widely shared media items about the environment in 2016. It identified comments trying to justify livestock production by comparing it to other (environmental) issues. Justifications were made by comparing livestock production with e.g. flying, and by comparing Sweden, perceived as an environmentally friendly country, to other countries which would engage in more environmentally detrimental actions. The study also found that doubting the quality of the information and information sources was used as a justification too (Olausson, 2018).

Given the little knowledge available in this field, this study aims to help to fill the gap by looking in detail at how the media covered this issue over a twelve-year period from 2006 to 2018. Based on an analysis of legacy media from the US and the UK, it seeks answers to the following groups of research questions: First, volume of coverage: how did it evolve over time? Were there significant differences between the US and UK, and between right-leaning and left-leaning media organizations? Second, causes and responsibilities: which sectors were mentioned the most in terms of being responsible for creating the demand for, or supply of, animal agriculture products. Which causes (seen as the processes which lead to GHG emissions) were most mentioned? And third, solutions: which solutions to reducing GHGs from animal agriculture were most mentioned?

## Method and research design

In order to address these research questions, this study conducted a quantitative media content analysis. This method is used by communication scholars to collect and analyze data about e.g. topics, actors, frames and biases present in mass media coverage or other communication material (Krippendorff, 2004). Online and print articles were analyzed from two media organizations (one left-leaning, one right-leaning) each in the UK and the US: The Guardian, the Telegraph, the New York Times and the Wall Street Journal, from January 1, 2006 to December 31, 2018. The start date was chosen to include any coverage of the FAO's report “Livestock’s Long Shadow”. This seminal 408-page report was released in November 2006. It identified livestock production as a major contributor to ecological destruction, including its large greenhouse gas emissions: “The livestock sector emerges as one of the top two or three most significant contributors to the most serious environmental problems, at every scale from local to global” (FAO, 2006, p. xx).

The selection of the US and the UK as the subjects of this study was made following a most-similar systems design (Esser & Vliegenthart, 2017); in other words, although there are important differences between the two countries particularly in the role of public sector broadcasting (US weak, UK strong), there are significant similarities in terms of its liberal media system (Hallin & Mancini, 2012), the presence of legacy media organizations with a worldwide presence in English, a politically polarized media environment, and declining print circulation being replaced (partially) by strong online presence, often accessed via social media (Newman et al., 2018). In addition, US and UK media titles are influential amongst policy makers outside of their home countries (O’Neill et al., 2015). The US and UK both have high per capita GHG emissions, and are influential players in international climate change negotiations (Boykoff, 2010). Climate change is subject to high levels of contestation and skepticism in both the media and public opinion in both countries (Painter, 2014; Painter & Ashe, 2012).

All four newspapers examined have a strong online presence aimed at a domestic and international market. In 2018 the Guardian online (15% of those surveyed) and the Telegraph online (7%) were the most used left-leaning and right-leaning up-market news sites respectively in the UK; in the same year in the US, the New York Times (9% of those surveyed) and the Wall Street Journal (5%) were among the four most consumed print newspapers in 2018, along with the Washington Post and US Today (Newman et al., 2018). The four studied titles score highly or relatively high in surveys regarding both levels of trust and weekly use amongst legacy media for their general news coverage, compared to other titles (Newman et al., 2018). The studied titles also entertain active social media accounts and have high follower numbers on Facebook (followers: New York Times 17 million, The Guardian 8.3 million, The Wall Street Journal 6.4 million, The Telegraph 4.4 million as of May 2020), Twitter (New York Times 46.5 million, The Guardian 8.8 million, The Wall Street Journal 17.7 million, The Telegraph 2.8 million), and Instagram (New York Times 8.8 million, The Guardian 3.1 million, The Wall Street Journal 2.8 million, The Telegraph 663,000).

All four titles aim for an English-speaking international audience beyond their national roots. The Guardian opened a digital-only US newsroom in 2011 and several offices in Australia in 2014 as part of its strategy to reach a global digital audience. The Telegraph is the largest-selling quality newspaper in the UK, but unlike the Guardian it has a paywall which is one reason why it has a lower reach worldwide compared to the Guardian. For several years the New York Times has based its business strategy on building up its domestic and international digital readers and subscriptions, and reducing its reliance on advertising. In 2015, the Wall Street Journal launched new global print and digital editions in Europe and Asia, as part of its efforts to secure more readers amongst executive decision makers around the world.

Previous studies have shown that they all cover climate change issues regularly, although the Guardian and New York Times have more dedicated environmental reporters (O’Neill et al., 2015; Painter et al., 2016). The Guardian in particular prides itself on the amount of environment reporters and the volume of climate change coverage, including the launch of a campaign for its readers in 2015 called “Keep it in the Ground” aimed at fossil fuel divestment (Salvesen, 2018). The New York Times has fluctuated in terms of its resources dedicated to climate change, but since 2017 has re-invigorated its coverage with a larger team and in-depth, often visual, reporting. The Telegraph has counted on a regular environment correspondence for several years, and also an array of sceptic commentators in its opinion pages (Painter, 2011). Like the Telegraph, the reporting of climate change in the Wall Street Journal tends to follow the mainstream science, but the paper often gives voice to uncontested skeptical voices in its opinion columns (Painter & Ashe, 2012).

A clear indication of the relative volume of coverage of climate change in the four titles from 2000 to 2020 can be found in the charts assembled by Boykoff et al. (2020). These show that the New York Times has the highest coverage in the US, particularly since 2017, and the Wall Street Journal is lower but still significant (for example, nearly 500 articles in 2009). Similarly, these charts show that for the UK over the same period, the Guardian and the Times have the most coverage of the newspapers included in their sample. The Telegraph’s coverage is lower, but amongst the highest in the UK. Similarly, other research confirms that for the UK, for the period 2007-2011, the volume of coverage of climate change in the Guardian was the highest, followed by the Times and then the Telegraph (Painter & Gavin, 2016).

We also know that left-leaning legacy media in general cover climate change more than right-leaning ones (Carvalho, 2007; Howard-Williams, 2009), and in the case of the 2015 COP21 summit in Paris, by a factor of more than two (841 compared to 396) (Painter et al., 2016). We also know from previous studies that in general the UK media cover climate change more than the US media. For example, a detailed study of the amount of media attention in several countries between 1996 and 2010 showed that the Guardian and Times together consistently had a higher percentage of articles dedicated to climate change than that of the New York Times and Washington Post (Schmidt et al., 2013). The study by Boykoff et al. (2020), would suggest that this trend has continued since then, although direct comparisons are problematic.

The four titles score highly or relatively highly in surveys regarding both levels of trust and reach amongst legacy media for their general news coverage (Newman et al., 2018). In 2018 the Guardian online and the Telegraph online were the most used left-leaning and right-leaning up-market news sites respectively in the UK; in the same year in the US, the New York Times and the Wall Street Journal were among the four most read print newspapers in 2018, along with the Washington Post and US Today (Newman et al., 2018).

We focused our analysis on articles that discussed livestock and animal agriculture in general, and that made a strong connection to climate impacts. We define animal agriculture to include the breeding, raising and slaughtering of animals, and the derivation of animal milk and egg for human consumption. We sampled articles in the data base Factiva using the following search terms: Climate Change AND (meat OR livestock OR vegan* OR vegetarian* OR animal agriculture OR cow OR cows) OR Global Warming AND (meat OR livestock OR vegan* OR vegetarian* OR animal agriculture OR cow OR cows). Based on pre-sampling research, we chose to include the search terms “climate change”, “global warming”, “livestock” and “animal agriculture” and search terms that were commonly mentioned in the articles relevant to our research interest, namely “meat”, “vegan”, “vegetarian”, “cow” and “cows.” These search terms allowed us to include articles such as the Wall Street Journal (August 17, 2014) article entitled “The next targets of the climate change enforcers will be livestock and all Americans who eat meat.” (Lusk, 2014); The Guardian (June 4, 2009) article stating that “Cows have digestive bacteria in their stomachs that cause them to belch methane, the second-most-significant heat-trapping emission associated with global warming after carbon dioxide.” (Kaufman, 2009); and The New York Times article making the statement: “With industrial-scale farms that each house thousands of cows, the region is also the center of a global debate about dairy’s impact on the environment.” (Gardiner, 2015); and The Guardian (April 1, 2018) article entitled “The unstoppable rise of veganism: how a fringe movement went mainstream” (Hancox, 2018).

Using these search terms Factiva gave us 5515 articles for the time-period January 1, 2006 to December 31, 2018. Articles that mentioned the connection of animal agriculture and climate change in only one sentence were excluded because articles with a stronger focus on the issue are more likely to leave a lasting impression on the reader. We also did not analyze articles which dealt with the impact of climate change on (animal) agriculture, such as reduced or increased productivity, changing weather patterns or hotter temperatures. After excluding these and other articles on the basis of irrelevance or repeats, we arrived at a total sample of 188 articles, of which 27 were from the New York Times, 7 from the Wall Street Journal, 113 from The Guardian and 41 from the Telegraph (see Table 1). Given the large number of articles captured by our search terms and the exclusion of 5327 irrelevant articles, and after additional searches with the inclusion of additional search terms, we observed that adding additional search terms, such as “cattle”, “beef” or “sheep” would not have added significant value to the sample. The final choice of search terms gave us a sample sufficiently robust and highly relevant to answer our research question of how animal agriculture and its impact on the climate are covered by the media.

**Table 1.** Sample.

	New York Times	Wall Street Journal	The Guardian	The Telegraph
2006	1	0	2	0
2007	2	1	3	7
2008	4	1	8	4
2009	3	2	24	4
2010	1	0	6	4
2011	2	0	3	1
2012	3	0	3	3
2013	1	0	10	0
2014	1	2	7	5
2015	2	0	15	4
2016	1	1	5	5
2017	2	0	10	1
2018	4	0	17	3
	27	7	113	41

For the elaboration of the codebook, we followed a mixed method approach of first drawing inductively on the relevant literature for each of the research areas (responsibilities, causes and solutions) to arrive at a preliminary list of variables. These were then refined deductively on the basis of an initial sample of articles. More details of the variables can be found in the discussion of the results below.

The 188 articles were coded by two coders who were trained extensively. The intercoder reliability test was conducted using 30 articles from all four news sources taken from across the whole studied time span. The intercoder reliability coefficient *Lotus* (Fretwurst, 2015) shows for all variables in the codebook, a standardized Lotus (S-Lotus) value of 0.86 and a non-standardized Lotus (Lotus) value of 0.93, which can be interpreted as 86% and 93% agreement between the coders. We were aiming to reach at least 80% agreement with the S-Lotus on all variables, and were under certain circumstances prepared to accept variables that did only reach this value of the Lotus. As for the Lotus values, we only had three variables that did not reach 80% agreement and none of those are reported in this study. We included the following variables with lower than wished for S-Lotus values: cause variables “Emissions from supply chains & distribution” (Lotus 0.83, S-Lotus 0.67), “Land use” (Lotus 0.83, S-Lotus 0.67), “Growing feed crops” (Lotus 0.83, S-Lotus 0.67) and “Greenhouse gases” (Lotus 0.8, S-Lotus 0.6); the responsibility variables “Consumers” (Lotus 0.77, S-Lotus 0.53) and “Factory farms” (Lotus 0.83, S-Lotus 0.67); and the solution variable “Meat consumption reduction” (Lotus 0.8, S-Lotus 0.6). Those variables were included because they still reached a sufficient Lotus value.

## Volume of media coverage

To give a general context for our results, we used Factiva and the search terms Climate Change OR Global Warming to estimate how the volume of coverage of animal agriculture and climate change compares to general climate change coverage from 2006 to 2018. None of these additional searches were filtered out to exclude irrelevant articles or doublets, so these numbers are mere estimates and only indicative. Using the search terms Climate Change OR Global Warming resulted in nearly 114,000 articles, and using the search terms mentioned above to capture animal agriculture and climate change resulted in a little over 5000 articles. This means that only about four per cent of the total number of articles about climate change included mention of animal agriculture.

**Figure 1.**
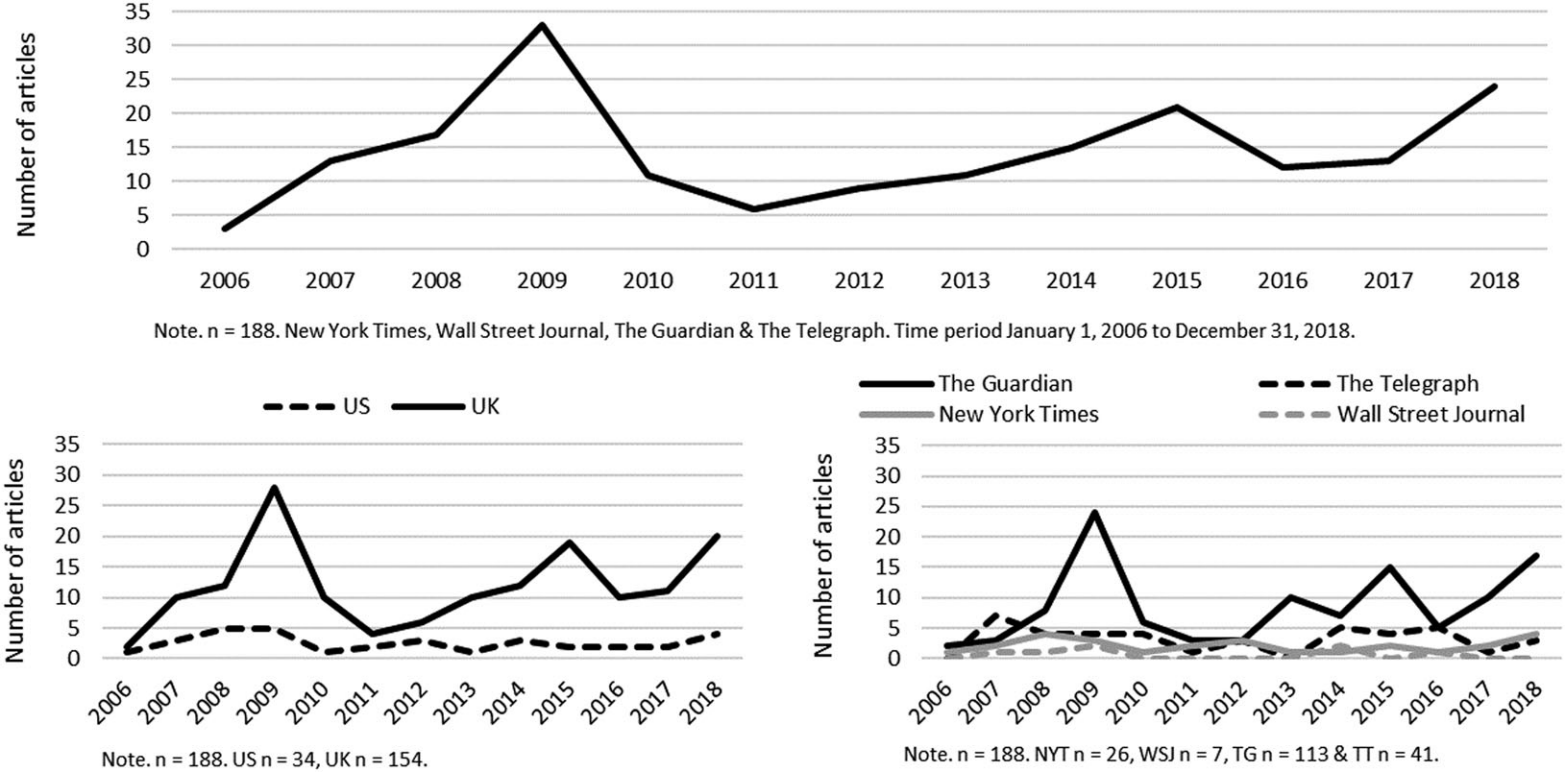
Volume of media coverage, by country, year and media organization.

Figure 1 gives the results for the total volume of coverage, broken down by country, then by newspaper. The most notable result seen in Figure 1 is that the peaks in 2009, 2015 and 2018 are mostly driven by increased attention from the Guardian. Indeed, as can be seen from Figure 1, whereas the coverage of the three other newspapers remains largely flat, that of the Guardian peaks in those years. Delving more into the drivers of these peaks, one can see that a large proportion of the coverage (24 articles) in 2009 was not, perhaps surprisingly, due to the reporting on the UN climate change summit in December in Copenhagen, which we know was a major driver of international coverage of climate change (Schäfer et al., 2014). Rather, an article initially published on 27 October quoting Lord Nick Stern, author of the 2006 Stern Report on the economics of climate change, on the need to reduce meat consumption to help the climate (Batty & Adam, 2009) prompted several commentary pieces on the same topic. Another driver was coverage of the Greenpeace report blaming British supermarkets for contributing to the destruction of the Amazon by using meat from farms responsible for illegal deforestation (Adam, 2009).

For the upturn in 2018 (17 articles), much of the Guardian’s coverage was based on the publication of eight journal articles and five NGO reports on the general topic of animal agriculture’s contribution to GHG emissions, often linked to the discussion of possible solutions at government policy level or individual behavior change. Many of these journal articles were covered in the Guardian online with links to the original publications. For example, the study of the environmental impact of more than 38,000 commercial farms in 119 countries by Poore and Nemecek (2018) appeared on the front page of the Guardian on 31 May, with the headline “Avoiding meat and dairy is “single biggest way” to reduce your impact on Earth” (Carrington, 2018). The article was liked, shared, or commented on more than a million times on Facebook (Buzzsumo, 2019), which is a very high figure for any of the paper’s articles on the environment. Similarly, Springmann et al. (2018) and Godfray et al. (2018) also received prominent coverage.

There is a sharp difference in the volume of coverage between left-wing titles (Guardian *n* = 113, plus New York Times *n* = 27, giving a total of *n* = 140) and right-wing titles (The Telegraph *n* = 41, plus WSJ *n* = 7 giving a total of *n* = 48). This is partly explained by climate change in general being seen as a left-wing issue of more interest to readers of left-leaning publications than a right-wing issue, particularly in countries like the US and UK where, as we have seen, climate skepticism is common both in the population and in the media and where views are often polarized along political lines (Painter & Ashe, 2012). These results are consistent with previous research which shows that coverage is greater in left-leaning news outlets, and in the UK compared to the US.

## Media discussion of responsibility

A list of the coded variables was initially drawn up on the basis of the research literature on the different causes or drivers of the contribution that animal agriculture makes to climate change, and for the different actors responsible for that contribution (in particular, FAO, 2006, 2018; Garnett et al., 2017; Gerber et al., 2013; Reisinger & Clark, 2017; Springmann et al., 2018). By the former, we meant the processes contributing to changes in the atmosphere and the resulting climate change impacts, and by the latter we meant those sectors of the population with agency in the processes.

For responsibilities, we identified several sectors responsible for climate emissions from the animal agriculture sector such as businesses (like Cargill and Tyson who produce animal products), consumers (who buy and consume them), supermarkets (who sell them), and governments or regulators (who debate, pass or enforce legislation governing the sector). We were aware that the livestock industry contains a broad range of farm size, production systems or methods, types of livestock, and huge country variations. However, journalists are often compelled by the nature of their trade to simplify complexity. So in our case study, the articles often refer to “businesses”, “farmers” or “factory farms” without specifying their essential characteristics or differences. After analyzing some of the newspaper articles, we arrived at eight variables, namely consumers, supermarkets, businesses, farmers, factory farms, governments, regulators and campaign/interest groups.

Table A gives examples from the sample of the first six type of actors often mentioned in the articles from different newspapers. “Businesses” means named companies such as Tyson or Cargill, “factory farms” is the generic term for large-scale intensive farming, whilst “famers” is the generic term for farmers or the farming sector without specifying farm size or type of production system.

**Figure 2.**
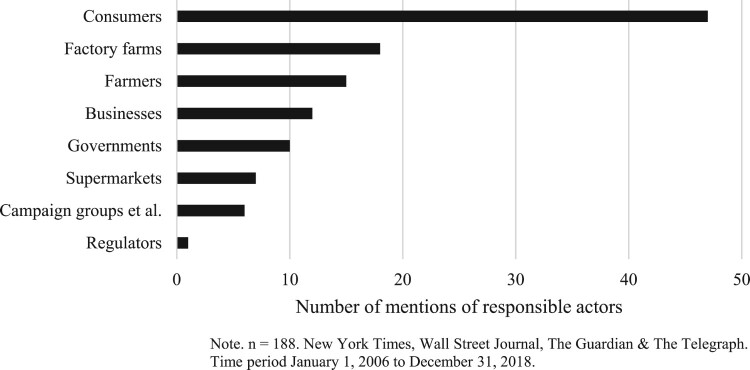
Mentioned responsible actors.

As can be seen in Figure 2, consumers are the most mentioned by some margin, namely 47 times in the 188 articles, well over twice as much as factory farms (18 times), farmers in general (15), and businesses (12). The other four sectors came in at 10 mentions or under. The same article can include mentions of more than one responsible actor.

**Figure 3.**
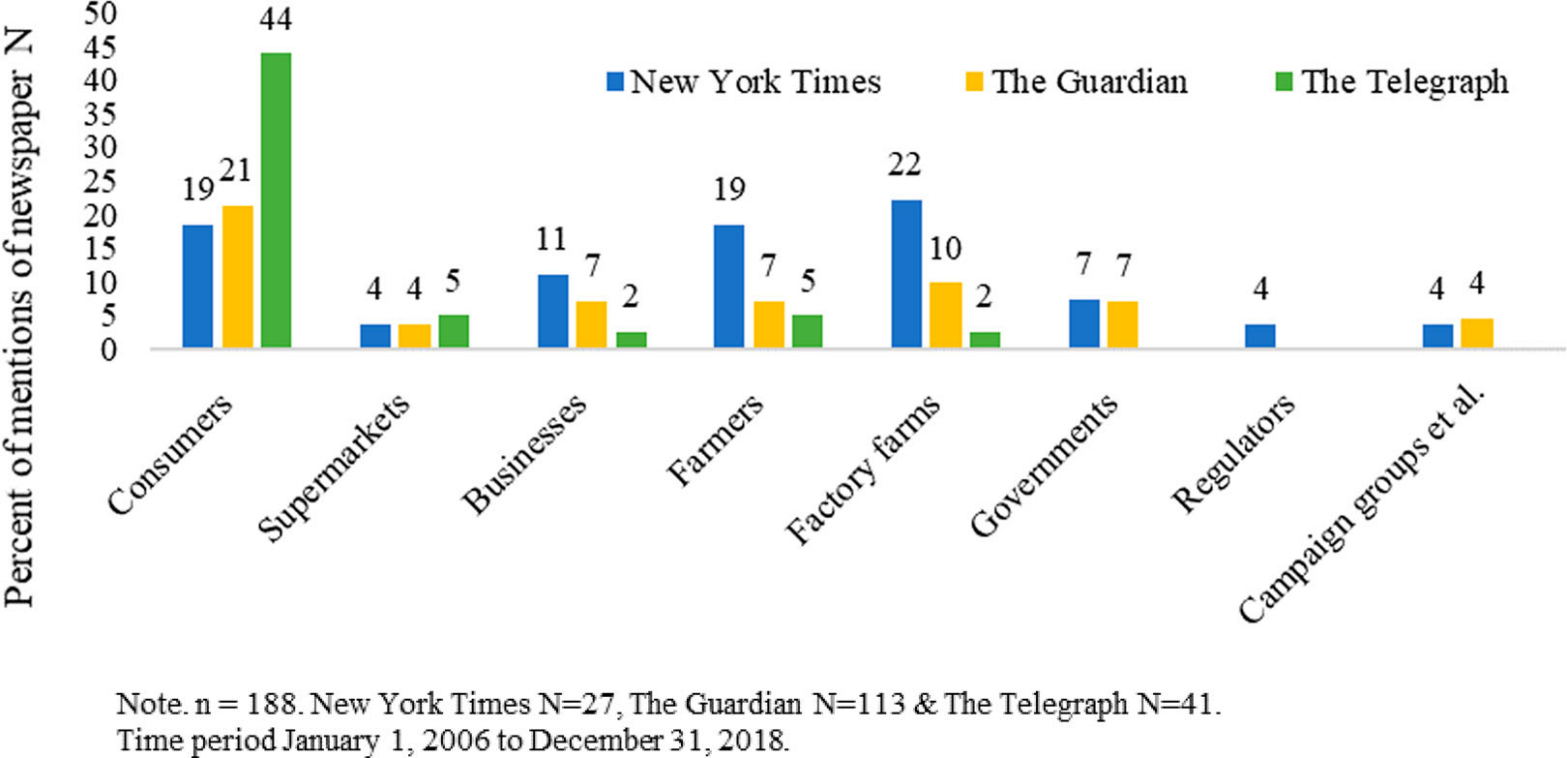
Mentioned actors by newspaper.

Figure 3 shows the results broken down by newspaper, expressed as a percentage of the total number of articles. In 19% of all New York Times articles consumers were mentioned as responsible actors, compared to 21% in the Guardian and 44% in the Telegraph. In contrast, businesses and factory farms were named in 41% of the New York Times articles, 17% of the Guardian, and only 7% of the Telegraph. There were no responsible actors mentioned in the Wall Street Journal.

It is interesting to note that consumers are mentioned most frequently as a responsible actor, and much more than governments or businesses. Some NGOs have long argued that large food and fertilizer companies such as JBS, Tyson, Cargill, and Yara do not attract the same public attention as fuel and gas companies like BP or Shell, given the size of their emissions. For example, the NGO Global Justice Now suggested in 2015 that Cargill for example, the largest beef producer in the US, had emissions comparable to the combined national emissions of Denmark, Bulgaria and Sweden, when all direct and indirect emissions from livestock production were included (Global Justice Now, 2015). In similar fashion, a 2018 study by three NGOs calculated that three companies, JBS, Tyson and Cargill, together emitted more GHGs in 2016 than France (GRAIN; IATP; Heinrich Böll Foundation, 2017). If the 20 largest meat and dairy companies were a country, the study suggested, they would have been the seventh largest GHG emitter in 2017, emitting more than Germany, Europe’s largest “climate polluter.”

We followed a similar method (deductively from the literature, inductively from the newspaper article) to draw up a list of variables for the causes of changes to the atmosphere as a result of livestock production. As mentioned above, the literature suggests that around 15% of global greenhouse emissions come from livestock production, 80% of which come from cattle and other ruminants (Garnett et al., 2017). Researchers often distinguish between direct emissions from livestock production, which are normally calculated to contribute about 10-12% of current global GHG emissions (Frank et al., 2019; Reisinger & Clark, 2017; Smith et al., 2014), and indirect emissions associated with livestock production such as land-use change and deforestation, energy use and animal feed production, which increase the total percentage figure to about 15% (Gerber et al., 2013). Methane release from enteric fermentation from ruminants is calculated to contribute about 40% of livestock GHGs, manure-related emissions around 25%, animal feed production about 13%, land-use change including land clearing and deforestation nearly 10%, and processing and transport from farm to retail about 3% (Gerber et al., 2013). Nitrous dioxide is released from manure and the use of fertilizers. It is worth stressing again that there is a “huge range in the emissions intensity of animal production, with variations by production system, agro-ecological context and management regime” (Garnett et al., 2017, p. 27).

Working from this literature and also from the newspaper articles, we defined 26 cause variables that broadly can be divided into different types: (i) either generic or specific gas emissions, and pollution (which journalists sometimes use as shorthand for greenhouse gas emissions), (ii) emissions from manure/slurry, fertilizer or pesticide usage, nitrogen runoff, (iii) emissions from land use changes including deforestation, (iv) emissions from growing feed crops for animals, (v) emissions from supply chains and distributions, (vi) population growth, growing demand for meat, and human diets and (vii) others. Examples of some of the most mentioned causes can be found in Table B.

**Figure 4.**
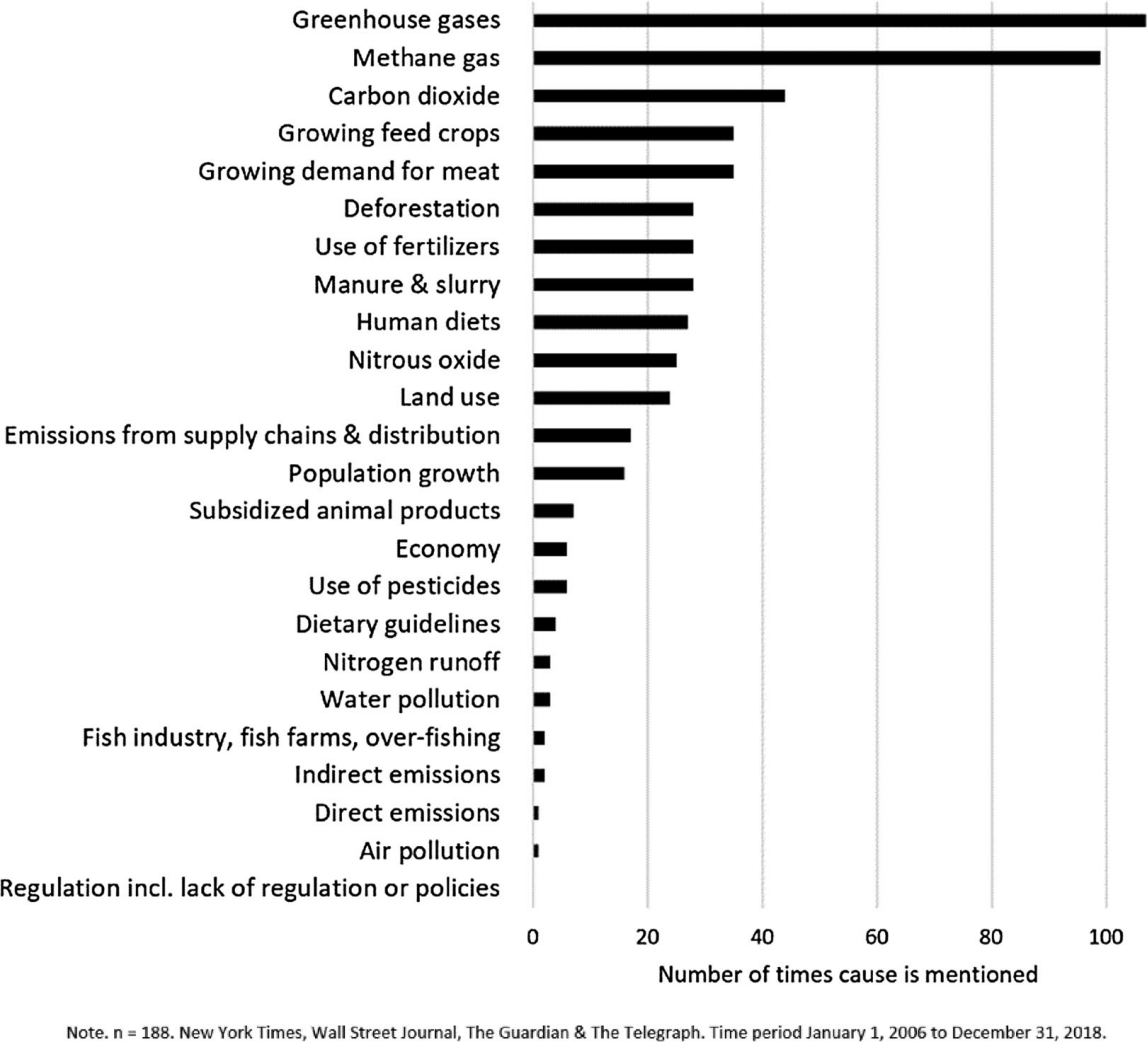
Mentioned causes.

Figure 4 gives the breakdown of the number of mentions by cause. As can be seen, greenhouse gases in general and in particular (methane, carbon dioxide and nitrous oxide) are mentioned over 100 times in our sample. Causes linked more indirectly to animal food production like growing feed crops (35), use of fertilizers (28), manure and slurry (28), land use (24), and emissions from supply chains and distribution (17) were less prominent. Wider social drivers such as growing demand for meat in many countries, human diets and population growth also featured, but with fewer mentions. It would be possible to conclude that the emissions from animal agriculture are seen much more as a problem of direct emissions from the digestive process of cows and other ruminants (burping, belching and farting) rather than indirect emissions from animal agricultural production systems (animal feed, fertilizer, energy, transport, and land use change). This reflects reality, as most of the GHGs from animal agriculture come from ruminant enteric fermentation (Ranganathan et al., 2016, p. 35).

## Solutions

Following a similar method, an initial list of four broad categories of solutions to animal agriculture’s greenhouse gas emissions was drawn up, based on the academic literature (Godfray et al., 2018; Harwatt et al., 2017; Poore & Nemecek, 2018; Springmann et al., 2018; Wynes & Nicholas, 2017). These were (a) consumer behavior change; (b) changes to agricultural practice such as technical innovations and management; (c) government policy or regulatory changes and (d) alternative protein sources. Examples of consumer behavior change included meat consumption reductions, veganism, vegetarianism, and eating other animal products with lower footprint. Changes to agricultural practice included a wide range of options such as grass-fed cattle, animal breeding such as genetic modification to reducing belching and farting, and other animal feed crops. Policy options featured an end to animal subsidies, or taxes on meat. New dietary guidelines was another solution category. Finally, alternative protein sources included insects, plant-based alternatives, and “cellular” meat also known as “clean” or “lab-grown” meat.

Examples of how the most mentioned solutions appeared in the media outlets can be seen in Table C.

**Figure 5.**
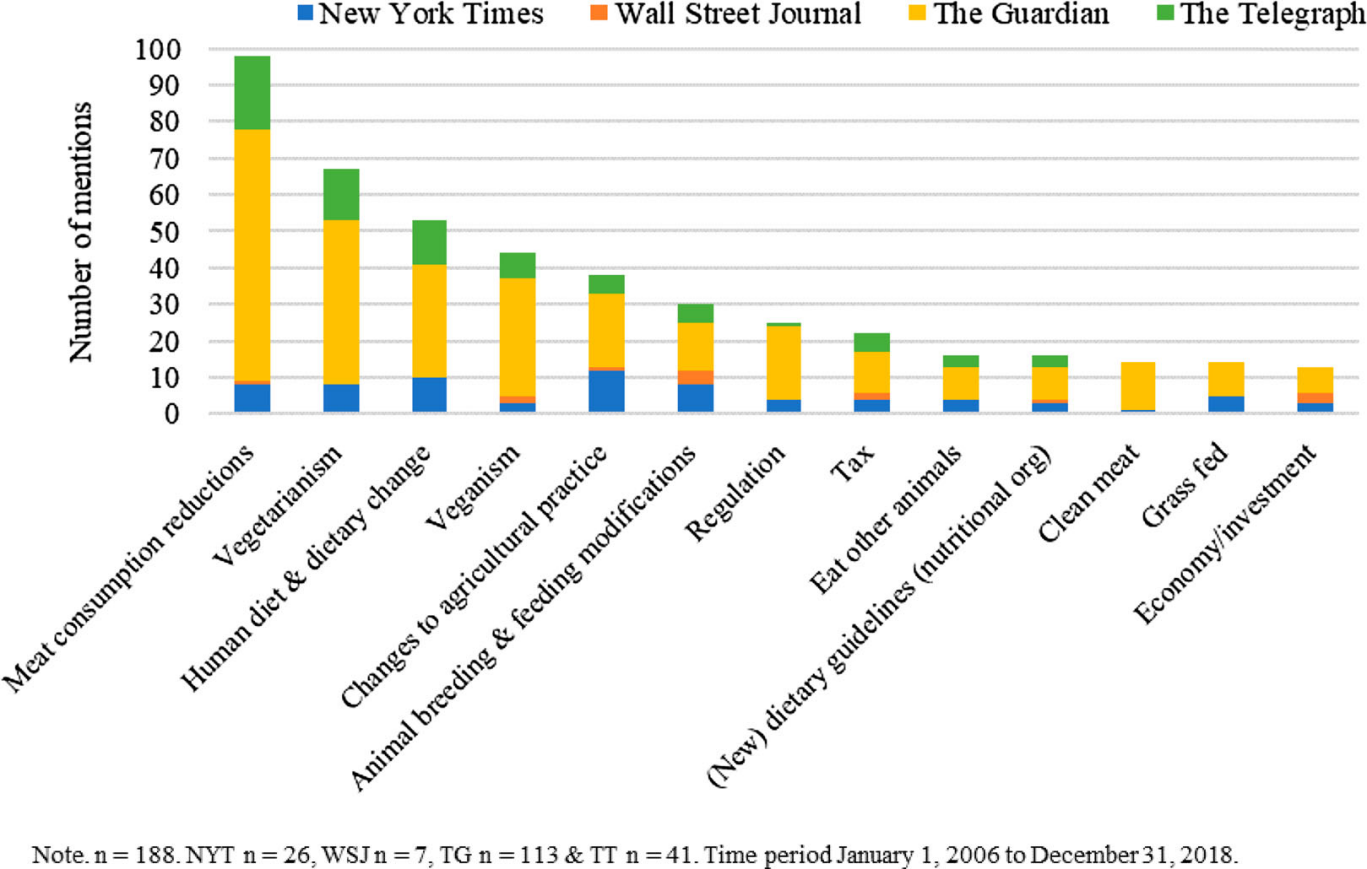
Mentioned solutions.

Figure 5 shows that (general) meat consumption reductions (such as the meatless Monday initiative) was the most prominent of the solutions with nearly 100 mentions, followed by vegetarianism (67), (general) changes to diet (53), and veganism (44). All four were particularly to be found in the Guardian. This was followed by changes to agricultural practice (38) and animal breeding/feeding modifications (30), and then regulation (25) and tax changes (22). Plant-based alternatives and/or clean meat were hardly mentioned.

**Figure 6.**
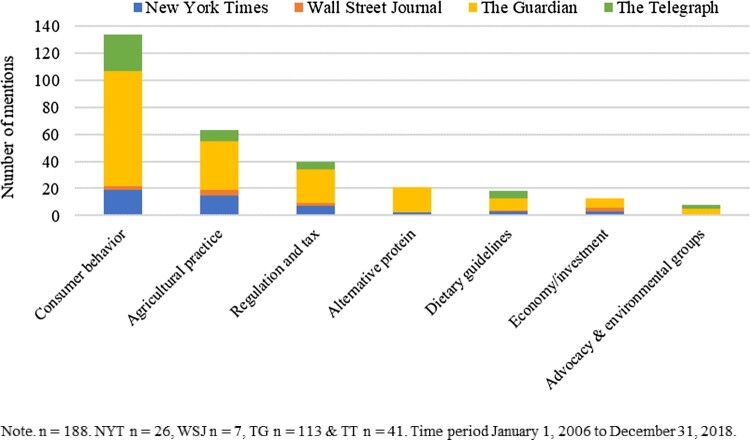
Categorized solutions.

Figure 6 shows the distribution of solutions appearing in the selected newspapers divided into seven main categories. It is clear that the overwhelming type of solution discussed in our media sample was variations on changes to individual consumption, rather than changes to the agricultural production methods, or regulation and tax. It is also of note that despite recent media and investment interest, plant-based and other alternatives to meat did not feature strongly in this sample as part of the solution to greenhouse gas emissions from the livestock sector.

## Conclusions

This study shows that from 2006 to 2018, animal agriculture’s contribution to greenhouse gas emissions did not feature strongly in the UK and US elite media’s coverage of climate change. This may have contributed to low public awareness of the link, as previous studies have shown that low volume of media coverage of climate change and food-related issues can play a (negative) role in public awareness of the issue and the importance the public assign to it, at least in the US, UK, China and Brazil (Happer & Wellesley, 2019).

The historically low amount of media coverage may in part be explained not by the absence of scientific studies on the topic, but by the apparent historical reluctance of sources often quoted by the media such as governments, politicians and environmental NGOs to advocate policies in this area. This may in turn be due to their belief (at least until recently) that the encouragement of dietary change is a harder “sell” to the public than changes to energy sources and transport means, or from a general reluctance to take on powerful lobbying interests in the farming sector.

The continuously low media attention given to this issue, as this study demonstrates, might also be a reflection of individual journalists’ views on climate change and mitigating solutions in general, or their and their media houses’ (conscious or unconscious) attitudes towards plant-based diets, their own eating habits and possibly even gender stereotypes. We are not aware of any studies examining this, but the vast majority of the human population eats animals, their milk and eggs, and it is likely this is no different for journalists. Pew’s study of American eating habits in 2016 showed that about 3% of Americans say that they follow a strict vegan or vegetarian diet, whereas an additional 6% say that they eat mostly vegan or vegetarian (Pew Research Center, 2016). Hence, as most people are, journalists too may be subject to the psychology of the “meat paradox” that enables people to care about animals at the same time as they eat them (Herzog, 2010; Joy, 2010; Loughnan et al., 2014; Singer, 1975).

Eating animals is also part of many people’s identity, and eating meat is tied to masculinity (Rothgerber, 2013). Newsrooms are still male dominated. According the Women’s Media Center, about 42% of people in US newsroom are women, and editors of the most widely distributed US newspapers are male (Women’s Media Center, 2019). Similar gender distribution numbers are reported from UK newsrooms (Green, 2019). This is certainly a question for future research, but it does not seem far-fetched that one factor explaining little media attention could be human eating habits in general and the unwillingness to change those habits.

Further studies are needed to assess the volume of coverage after 2018, and particularly the publication of the special IPCC report on *Climate Change and Land* in August 2019 (IPCC, 2019a), which discussed in detail the options for reducing GHG emissions from livestock production, including changes to agricultural systems and dietary options. All four of our selected media organizations reported extensively on this report (Carpani, 2019; Carrington, 2019; Hotz & Bunge, 2019; Sengupta, 2019), often concentrating on the IPCC observation that “balanced diets, featuring plant-based foods […] present major opportunities for adaptation and mitigation” (IPCC, 2019b, p. 24). In addition, the editorial initiative launched in February 2018 by the Guardian called “Animals Farmed” (The Guardian, 2018) (and supported by the Open Philanthropy Project) has increased the volume of media scrutiny of factory farming, food production, as well as animal welfare.

Research suggests that multi-scale approaches at both the individual and state levels will be needed to reduce greenhouse gas emissions (Ostrom, 2012). In the area of animal agriculture, changes in individual dietary behavior are also seen as a very important component of the solution mix (Springmann et al., 2018). However, a complex array of policy actions will also be needed to change food supply and production systems, particularly to make agriculture support better diets within environmental boundaries (Parsons & Hawkes, 2019; Willet et al., 2019). For example, new dietary guidelines alone are likely to be less effective: US dietary guidelines have recommended at least five servings a day of fruit and vegetables but consumption does not reflect recommendations as the vast majority of Americans do not consume that amount (Centers for Disease Control and Prevention, 2017). Greater media discussion of the array of available solutions would help restore the balance away from only the individual consumer being responsible. Some of these solutions are accessible without the development of complex technologies and include refrigerant management, reduced food waste, plant-rich diets, educating girls and regenerative agriculture (Project Drawdown, 2019).

This media emphasis on individual consumer behavior chimes with a general trend towards “individualization” of environmental responses, particularly in the US, where responsibility for environmental damage is seen by some scholars to have shifted onto individuals and so solutions must come from individuals too (Maniates, 2001). But as Maniates argues (p.31), “When responsibility for environmental problems is individualized, there is little room to ponder institutions, the nature and exercise of political power, or ways of collectively changing the distribution of power and influence in society.”

In a similar vein, some also argue that a strong (media) focus on individual consumer choices helps to take the responsibility off the necessary systemic changes that governments should be introducing, and distracts attention from the holding to account of large (polluting) corporations (Mann & Brockopp, 2019).

As our results show, government regulation or taxes were mentioned in our media sample much less than consumer solutions, a result which is mirrored in government responsibility being mentioned five times less than consumer responsibility. As our results also show, the combined mention of businesses and factory farms as responsible for animal agriculture’s contribution to emissions is well below the total number of mentions of consumers. The media pay less attention to suppliers and more on the demand makers, the consumers.

It might seem like a contradiction that so much emphasis is put on individual change yet at the same time veganism is the least discussed solution of the four discussed individual changes (meat consumption reduction, vegetarianism, human diet and dietary change and veganism). Whereas all these individual actions are a step in the right direction to help lessen greenhouse gas emissions, it is worthy of further research to address the question of why what has been seen as the most effective of these options, namely a vegan diet, is the least discussed individual action. According to a widely quoted study by Poore and Nemecek (2018), adopting a diet that excludes animal products can reduce food’s greenhouse gas emissions by 49%, land use by 76%, acidification by 50%, eutrophication by 49% and scarcity-weighted freshwater withdrawals by 19%. The authors calculate that if consumption of each animal product was reduced by 50%, this would achieve “71% of the previous scenario’s GHG reduction […] and 67, 64, and 55% of the land use, acidification, and eutrophication reductions” (Poore & Nemecek, 2018, p. 5).

Linking our findings to the wider literature on interest group influence, and particularly that of food and meat companies on media outputs, would also be a fruitful future research direction. For example, Almiron et al. (2018) have shown how the media system is influenced by the economy, the political system and society in general, and specifically how the “(mis)treatment of other animals is the result of public consent supported by a morally speciesist-anthropocentric system of values” and is largely a neglected issue (Almiron et al., 2018, p. 367). The same authors’ discussion of the promotion of certain food products by powerful sectors in the food industry is apposite here, as they show how the promotion of fast food and animal-based product not only increases revenue, but also promotes the “culture” of eating certain foods to help preserve those who offer such foods (Almiron et al., 2018). Research from Australia also shows that unhealthy foods are heavily promoted by food companies sponsoring elite sport broadcast by the mass media (Dixon et al., 2018).

In our results on causes and solutions, the farming sector is also much less mentioned than consumers. This may be an indication of a relative lack of media scrutiny of large-scale, intensive animal farming, which is linked to the draconian “ag-gag” laws in some states in the US which put strong restrictions on reporting on what goes on in such farms (Ceryes & Heaney, 2019). It also may be due to a general lack of resources for in-depth investigative reporting of this sector, although the Guardian initiative on “Animal Farmed” has helped to fill the gap. At the same time, there is no lack of animal rights and liberation activists’ and advocates’ visual content from the inside of farms and slaughterhouses on social media. More research is needed as to why these sorts of images do not seem to be picked up regularly by mainstream media.

More qualitative interview work with journalists and editors could shed light on the obstacles to more reporting of the links between animal agriculture and its contribution to climate change, the reasons behind the strong focus on individual behavioral change, and the lack of scrutiny of large animal food businesses (in contrast to the amount of media attention paid to major US and UK oil and gas companies). Such a qualitative approach could also scrutinize the lack of detailed media discussion on the wider-reaching consequences of changes towards a more just food system, in the same way that economic growth and justice opportunities are often mentioned in the coverage of energy system transformations (public health, green jobs, and equity issues).

## Appendices

Table A. Examples of responsibilities.

**Table:**

Consumers	“People in rich countries should limit their meat-eating to the equivalent of one hamburger per person per day to help stave off global warming, a study published yesterday suggests”. (The Telegraph, September 14, 2007)
Supermarkets	“While the focus of the findings were on global agri-food businesses, Tara Garnett, coordinator of the food climate research network at Oxford University, says the responsibility for climate emissions lies across the food chain with consumers who eat the food and supermarkets who sell it, as well as manufacturers like Cargill and Tyson who produce it”. (The Guardian, December 7, 2015)
Businesses	“While the focus of the findings were on global agri-food businesses, Tara Garnett, coordinator of the food climate research network at Oxford University, says the responsibility for climate emissions lies across the food chain with consumers who eat the food and supermarkets who sell it, as well as manufacturers like Cargill and Tyson who produce it”. (The Guardian, December 7, 2015)
Farmers	“The UK farming sector only accounts for around 1% of the country’s total CO_2_ emissions, and methane emissions from UK agricultural production have fallen by 17% since 1990”. (The Guardian, November 3, 2009)
Factory Farms or large-scale farms	“With industrial-scale farms that each house thousands of cows, the region is also at the center of a global debate about dairy’s impact on the environment. […] Globally, dairy production accounted for 2.8% of all man-made climate-warming gases in 2005”. (New York Times, May 1, 2015)
Governments	“Zohra Bouamra-Mechemache, a co-author of the French study, readily acknowledged that the proposed carbon tax on beef has no chance of becoming reality, “not even in Europe” and certainly not in the United States. Our politicians continue to regard the beef industry as, well, a sacred cow”. (New York Times, March 18, 2018)

Table B. Examples of causes.

**Table:**

Greenhouse gases (unspecified)	“The original version of the film claimed 51% of global greenhouse gases were produced by animal agriculture, based on a single, non-peer-reviewed academic paper – the scientific consensus is closer to 15%”. (The Guardian, May 2, 2018)
Methane gas	“Methane is among the most potent greenhouse gases, and researchers now believe livestock industries are a major contributor to climate change, responsible for more greenhouse-gas emissions than cars are, according to the United Nations”. (Wall Street Journal, February 26, 2009)
Carbon Dioxide	“In a paper published last month in the American Journal of Clinical Nutrition, food ecologist Annika Carlsson-Kanyama showed that kilo for kilo, beef and pork could produce 30 times more CO_2_ emissions than other protein rich foods such as beans”. (The Guardian, May 16, 2009)
Growing feed crops (for animals)	“United Nations figures suggest that meat production is responsible for about 18% of global carbon emissions, including the destruction of forest land for cattle ranching and the production of animal feeds”. (The Telegraph, October 27, 2009)
Growing demand for meat	“But the really serious issue is the intensive farming of livestock, particularly cows and sheep, which generate as much as a half of the total emissions. […] And, worldwide, the gradual increase in the consumption of meat creates pressure to cut down forests to create new pastureland and cropland for grains to help feed the livestock”. (The Guardian, October 27, 2009)
Use of fertilizers	“Methane is produced by the digestive processes of livestock, while nitrous oxide is released by many agricultural fertilisers. Scientists say both contribute to global warming, along with carbon dioxide (CO_2_)”. (The Telegraph, July 8, 2008)
Deforestation	“Deforestation to make way for livestock, along with methane emissions from cows and fertilizer use, creates as much greenhouse gas emissions as all the world’s cars, trucks and airplanes”. (The Guardian, December 21, 2018)

Table C. Examples of solutions.

**Table:**

Meat Consumption Reductions (general)	“Growing food for the world’s burgeoning population is likely to send greenhouse gas emissions over the threshold of safety, unless more is done to cut meat consumption, a new report has found”. (The Guardian, March 21, 2016)
Vegetarianism	“A recent study by Oxford University found that meat-rich diets – defined as more than 100 g of meat per day – resulted in around 7.2 kg of emissions. In contrast, both vegetarian and fish-eating diets caused about 3.8 kg of CO_2_ per day, while vegan diets produced only 2.9 kg”. (The Telegraph, October 2, 2016)
Human diet and dietary change	“Adopting healthier and more environmentally sustainable diets can be a large step in the right direction”. (The Guardian, March 21, 2016)
Veganism	“A recent study by Oxford University found that meat-rich diets – defined as more than 100 g of meat per day – resulted in around 7.2 kg of emissions. In contrast, both vegetarian and fish-eating diets caused about 3.8 kg of CO_2_ per day, while vegan diets produced only 2.9 kg”. (The Telegraph, October 2, 2016)
Changes to agricultural practice (general)	“By focusing on making agriculture more efficient and encouraging people to reduce the amount of meat they eat, we could keep global temperatures within the two degrees threshold”. (The Telegraph, June 20, 2012)
Animal breeding and feeding modifications	“We may be able to reduce our impact on the environment by eating less meat, but we can also do the same by using science to make livestock more productive and environmentally friendly”. (The Wall Street Journal, August 17, 2014)
Regulation	“Tobias Baedeker, of the World Bank, said farmers would require a lot of support to make the changes required but that redirecting the world’s huge subsidies could be a ‘game-changer’”. (The Guardian, December 5, 2018)
Tax	“Some industry critics have even called for new “meat taxes” to discourage consumption”. (The Wall Street Journal, August 17, 2014)
